# Application and Development of Cell Membrane Functionalized Biomimetic Nanoparticles in the Treatment of Acute Ischemic Stroke

**DOI:** 10.3390/ijms25158539

**Published:** 2024-08-05

**Authors:** Ying Li, Chuang Wu, Rui Yang, Jiannan Tang, Zhanqing Li, Xue Yi, Zhongxiong Fan

**Affiliations:** 1 Xiamen Key Laboratory of Traditional Chinese Bio-Engineering, Xiamen Medical College, Xiamen 361021, China; 2 School of Pharmaceutical Sciences and Institute of Materia Medica, Xinjiang University, Urumqi 830017, China

**Keywords:** acute ischemic stroke, ischemic stroke, biomimetic nanoparticles, drug delivery system, cell membrane, pathophysiology

## Abstract

Ischemic stroke is a serious neurological disease involving multiple complex physiological processes, including vascular obstruction, brain tissue ischemia, impaired energy metabolism, cell death, impaired ion pump function, and inflammatory response. In recent years, there has been significant interest in cell membrane-functionalized biomimetic nanoparticles as a novel therapeutic approach. This review comprehensively explores the mechanisms and importance of using these nanoparticles to treat acute ischemic stroke with a special emphasis on their potential for actively targeting therapies through cell membranes. We provide an overview of the pathophysiology of ischemic stroke and present advances in the study of biomimetic nanoparticles, emphasizing their potential for drug delivery and precision-targeted therapy. This paper focuses on bio-nanoparticles encapsulated in bionic cell membranes to target ischemic stroke treatment. It highlights the mechanism of action and research progress regarding different types of cell membrane-functionalized bi-onic nanoparticles such as erythrocytes, neutrophils, platelets, exosomes, macrophages, and neural stem cells in treating ischemic stroke while emphasizing their potential to improve brain tissue’s ischemic state and attenuate neurological damage and dysfunction. Through an in-depth exploration of the potential benefits provided by cell membrane-functionalized biomimetic nanoparticles to improve brain tissue’s ischemic state while reducing neurological injury and dysfunction, this study also provides comprehensive research on neural stem cells’ potential along with that of cell membrane-functionalized biomimetic nanoparticles to ameliorate neurological injury and dysfunction. However, it is undeniable that there are still some challenges and limitations in terms of biocompatibility, safety, and practical applications for clinical translation.

## 1. Introduction

According to the 2019 Global Burden of Disease Study (GBD), stroke is the second leading cause of death and the third leading cause of disability worldwide [1]. Strokes are commonly classified into three categories: ischemic strokes, transient ischemic strokes, and hemorrhagic strokes, with ischemic strokes accounting for approximately 80% of all stroke cases, as reported in sources [2,3]. The limited ability of tPA to target thrombi and the short half-life of the drug as well as the narrow therapeutic time window for thrombolysis (4.5 h) somewhat limit the use of tPA. Ischemic stroke is identified as a neurological emergency, characterized by the death of brain cells due to ischemia resulting from the occlusion of intracranial arteries, supported by neuropathological, neuroimaging, and clinical findings [4]. Ischemic strokes are usually caused by a blood clot blocking an artery in the brain [3]. This blockage causes ischemia, hypoxia, and malnutrition in the brain, leading to severe consequences. These include the overproduction of cerebrospinal fluid (hydrocephalus), generation of reactive oxygen species (ROS), and release of inflammatory factors [5].

As per the latest guidelines from the American Stroke Association, the recommended standard treatment for acute ischemic stroke (AIS) involves early reperfusion through intravenous thrombolysis or mechanical thrombus retrieval [6]. At present, tissue plasminogen activator (tPA) is the sole FDA-approved thrombolytic agent for treating ischemic stroke [3]. The limited targeting capability of the thrombus and the short half-life of the drug contribute to a narrow time window for thrombolysis (4.5 h). Furthermore, intravenous administration of tPA poses the risk of hemorrhagic transformation (HT) [7]. If the patient misses the tPA administration window or if the clot persists, mechanical thrombectomy for physical removal of the clot is an option. Endovascular mechanical thrombolysis for proximal lesions offers advantages over intravenous thrombolysis, including a relatively extended therapeutic time window (6 h) and a higher revascularization rate [8]. However, mechanical thrombolysis demands precision and entails stringent requirements for both physicians and equipment. Consequently, only a limited subset of patients qualifies for endovascular mechanical thrombolysis in the clinical management of ischemic stroke [9,10]. In general, despite the advancements in intravenous and mechanical thrombolysis, only a small percentage of patients (2% to 7%) benefit from standard treatment. This limitation is primarily attributed to the narrow therapeutic time window, lack of awareness regarding stroke risks, and insufficient availability of associated medical equipment [6].

For most patients who surpass the optimal treatment window, focusing on neuroprotection and neurorepair becomes crucial to salvaging the ischemic penumbra (reperfused or partially perfused tissue surrounding the infarct core). This approach is especially significant in addressing the dire prognosis associated with acute ischemic stroke (AIS) [11]. Indeed, despite extensive research on neuroprotective agents, their efficacy in clinical trials has often ended in disappointment. This can be attributed to common shortcomings such as low solubility, short half-life, and poor blood–brain barrier permeability in vivo, which hinder the effective delivery of the drug to the lesion site [11,12]. The efficacy of treating AIS is influenced by the preserved integrity of the blood–brain barrier (BBB) and the intricate nature of the brain microenvironment. These factors pose significant challenges to delivering therapeutic agents effectively to the affected areas of the brain [13]. The BBB serves as a highly specialized neurovascular unit, acting as a protective barrier against toxins, pathogens, and potentially harmful substances from entering the brain. However, this protective function also presents challenges for effectively delivering drugs to the site of brain lesions. The BBB’s selective permeability makes it difficult for therapeutic agents to penetrate and reach their intended targets within the brain parenchyma [14]. Thus, researching drugs that can efficiently target and accumulate at stroke lesion sites is crucial for treating AIS.

Nanomedicine involves utilizing nanotechnology to either shrink the active ingredient of a drug into nanometer-sized particles or combine it with appropriate nanomaterials to create such particles [15]. Nanomedicines offer several advantages, such as their nanoscale size (100~500 nm), high encapsulation rate, larger surface area, controlled release properties, integration of diagnostic and therapeutic functions, and targeted payload delivery capability. These attributes make nanomedicine a promising approach for improving drug delivery and enhancing therapeutic outcomes [16,17]. Extensive research on nanodrug delivery systems in recent years has highlighted their role in traversing biological barriers and efficiently transporting drugs to lesion sites through multiple mechanisms, garnering significant attention. Targeted delivery remains the primary focus for such drug delivery systems. Passive targeting, often combined with active targeting, aims to optimize drug delivery to specific lesions, such as tumors, while minimizing off-target distribution. Active targeting involves modifying the surface of nanocarriers to recognize and bind to specific receptors through ligand–receptor interactions, enhancing the specificity and efficacy of drug delivery [6,10,15].

Biomimetic drug delivery systems, which incorporate components of biological cell membranes along with synthetic nanoparticles, indeed represent an innovative and increasingly researched approach in nanomedicine. These systems leverage the inherent properties of cell membranes to create carriers that mimic the natural interactions of cells within the human body. This biomimicry can significantly enhance the effectiveness of drug delivery. In these systems, active targeting is facilitated by modifying the surface ligands of nanocarriers to specifically bind to receptors on the target cells, increasing the precision with which drugs are delivered to diseased or damaged tissues. By employing cell-derived biomimetic carriers, these systems utilize the natural delivery mechanisms of endogenous cells, which can lead to improved delivery efficiency and therapeutic outcomes. Camouflaging synthetic nanoparticles with natural cells or cell membranes are promising strategies to evade the immune system’s mononuclear phagocyte system (also known as the reticuloendothelial system). This approach helps to reduce the immunogenicity of the nanoparticles, thereby decreasing the likelihood of an immune response against the delivery system and extending the circulation time of the drug in the bloodstream. This prolongation allows for more sustained drug release and enhanced therapeutic efficacy [18].

**Figure 1:**
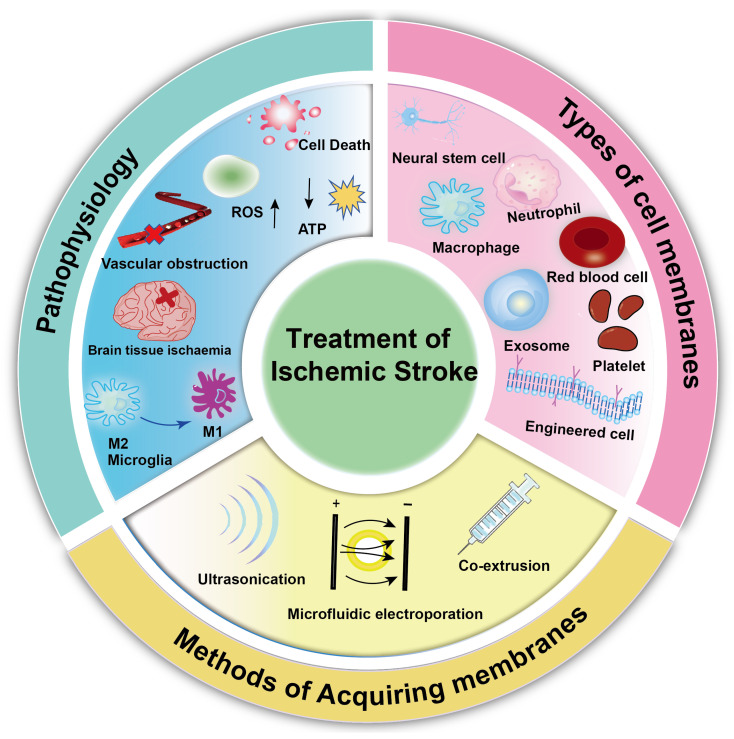
This figure provides a brief overview of this review, including stroke pathophysiology, methods of cell membrane acquisition, and types of bionic cell membranes.

Biomimetic nanoparticles have emerged as a promising solution for overcoming the challenges of drug delivery across the BBB in treating AIS. The unique properties of biomimetic nanoparticles, which combine components of biological cell membranes with synthetic nanoparticles, offer advantages in enhancing drug permeability across the BBB [16,19]. Biomimetic nano-delivery systems, such as platelet membrane-derived nanocarriers, hold the potential for targeted drug delivery to ischemic stroke sites, promoting vasodilation and reperfusion of micro-vessels. Natural platelets (PLTs) play a crucial role in adhering to damaged blood vessels during thrombosis. Nanocarriers modified with platelet membranes exhibit an excellent ability to target stroke lesion sites [20,21,22]. Erythrocytes, another vital component of human blood, are abundant, easily accessible, and subject to millions of rapid circulatory flows in the arterial vascular system. Their excellent plasticity and robustness of the erythrocyte membrane and cytoskeleton, combined with the absence of a nucleus and organelles and specific molecular features on the erythrocyte surface, render erythrocytes and their membranes ideal bionic nanocarriers [23]. Apart from platelets and erythrocytes, nanocarriers derived from cell membranes of neutrophils, macrophages, exosomes, neural stem cells, and engineered cells have demonstrated significant potential as therapeutic drug delivery systems for ischemic stroke (IS) [24]. These biomimetic nanocarriers can effectively decrease drug dosage, mitigate side effects, and offer precise drug release rates, significantly enhancing the therapeutic effectiveness against ischemic stroke. This paper provides a narrative overview of the pathophysiological aspects of ischemic stroke, outlines the preparation techniques of biomimetic nanoparticles based on current research trends in drug delivery, and highlights preclinical investigations involving various types of cell membrane-functionalized biomimetic nanoparticles for treating AIS (Figure 1). For this review, we searched PubMed, x-mol, NCBI (National Center for Biotechnology), and other databases to obtain the relevant literature on this topic in the last 5 years. It is only for readers’ reference.

## 2. Pathophysiology of Ischemic Stroke

Ischemic stroke is characterized by a complex interplay of mechanisms that together result in brain damage. This process involves intensified neuronal death and apoptosis due to insufficient blood flow and oxygen, which leads to the formation of penumbral regions around the core infarct zone. The acute phase reveals the dynamic nature of ischemic brain damage, as reduced oxygen and energy supply trigger stress responses. These responses increase levels of ROS and cytokines and activate microglia and astrocytes. The cytokines released by these glial cells compromise the integrity of the BBB and prompt peripheral neutrophils to infiltrate the brain, thereby amplifying the inflammatory response. Such inflammation leads to brain edema, further BBB damage, and neuronal death. During the chronic recovery phase, macrophages migrate to ischemic areas of the brain to facilitate neuronal repair and regeneration. Notably, abnormal excitability, inflammatory responses, oxidative stress, and widespread depolarization are significant contributors to cell death in this context.

### 2.1. Excitotoxicity

Stroke represents a complex pathological process driven by multiple factors and manifesting at various levels. A critical early event in this cascade is glutamate-induced excitotoxicity, occurring during the hyperacute phase of the cerebral ischemic response [25]. Once cerebral blood vessels become obstructed, there is an immediate cessation of oxygen, glucose, and other essential nutrient supplies to the ischemic area. This disruption causes a rapid decline in ATP production and subsequent depletion of energy reserves, which initiates an ischemic cascade. The energy crisis impairs ion pumps and activates voltage-dependent calcium channels. This activation triggers a presynaptic overload of calcium ions and the release of substantial amounts of glutamate into the synaptic cleft. Furthermore, due to the energy shortfall, glutamate is not effectively recycled by astrocytes and may even be released back into the synaptic cleft, further increasing its concentration in the synaptic gap. This excessive accumulation of glutamate leads to the overactivation of postsynaptic glutamate receptors, culminating in a postsynaptic overload of calcium ions and significant neurotoxicity [26].

### 2.2. Oxidative Stress

Oxidative stress is crucial in the pathogenesis of the ischemia-reperfusion process and exerts a detrimental effect on the development of post-stroke neurological dysfunction. In the early stages of this disruption in energy metabolism, additional ROS are produced. These ROS activate microglia, which express high levels of zinc-dependent proteases such as Matrix Metalloproteinases (MMPs), leading to the disruption of the BBB [27].

The disruption of the BBB is further exacerbated by several factors: impaired endothelial cell tight junctions, edema in the protruding endings of astrocytes, increased microglial inflammatory activity, and elevated levels of cytokines including Vascular Endothelial Growth Factor (VEGF), Nitric Oxide (NO), interleukin-6 (IL-6), interleukin-1β (IL-1β), and Tumor Necrosis Factor-alpha (TNF-α). Additionally, peripherally infiltrating inflammatory cells play a complex role; for example, regulatory T cells can exert an anti-inflammatory effect through interleukin-10 (IL-10), helping to protect the integrity of the BBB.

### 2.3. Inflammatory Response

In ischemic stroke, the obstruction or narrowing of cerebral blood vessels leads to a significant reduction in oxygen and nutrient supply to the brain, causing neuronal death. These dead nerve cells release a surge of cytokines, including inflammatory mediators and chemokines such as TNF-α and interleukin-1 (IL-1), which initiate an inflammatory response. In response to ischemic injury, neurons and astrocytes release additional inflammatory mediators and neurochemicals like glutamate, exacerbating damage to vascular endothelial cells and increasing vascular permeability. This sequence of events contributes to cerebral edema and the disruption of the blood–brain barrier [28].

Chemokines attract leukocytes to the ischemic region, where macrophages and astrocytes become activated, releasing more inflammatory mediators, and intensifying the inflammatory response. These mediators are toxic to peripheral nerve cells, further exacerbating nerve damage. Therapeutic strategies often involve the use of inflammation inhibitors such as glucocorticoids and non-steroidal anti-inflammatory drugs (NSAIDs). Additionally, inflammation-regulating proteins like transforming growth factor-beta (TGF-β) and interleukin-10 (IL-10) are employed to reduce nerve damage and facilitate nerve repair, aiming to suppress the inflammatory response and improve recovery outcomes in patients with ischemic stroke [29].

## 3. Progress of Research on Biomimetic Nanoparticles

### 3.1. Methods of Acquiring Cell Membranes

The process of obtaining cell membranes involves several crucial steps to ensure the quality and suitability of the membrane for a particular biomedical application. Initially, cells are cultured from appropriate cell types, selected based on their intended application (e.g., tumor cells for cancer treatment or immune cells for immunomodulation). These cells are cultured under controlled conditions until they reach the desired growth and density. Subsequently, the cells are lysed, either through mechanical methods such as ultrasonic treatment or chemical means like using Triton X-100 detergent. This lysis process releases the internal cellular structures. The resultant cell membranes are then isolated and purified using techniques such as centrifugation. Multiple rounds of centrifugation, potentially coupled with density gradient centrifugation, may be employed for further purification. To ensure functionality, the isolated cell membranes undergo processing to confirm the presence of functional membrane proteins, pivotal for the desired nanoparticle function. Finally, the purified cell membranes are bound to the nanoparticles using techniques like extrusion, co-incubation, or microfluidics. These methods facilitate the spontaneous wrapping of the cell membranes around the nanoparticle surfaces, thereby enhancing their biocompatibility and targeting properties. This process demands not only scientific complexity but also meticulous control of each step to guarantee the quality and efficacy of the final product [30].

### 3.2. Membrane Preparation of Biomimetic Nanoparticles for Cell Membrane Functionalization

Presently, the preparation of CMC@NPs (cell membrane-coated nanoparticles) predominantly employs a top-down technological approach. The process initiates with the extraction of cell membranes, achieved by disrupting the cellular membrane structure through methods like low-osmotic lysis, physical homogenization, or repeated freeze–thaw cycles. Following membrane disruption, the intracellular contents are extracted using differential centrifugation, facilitating the collection of membrane sheets.

If necessary, these membrane sheets can be resized to the desired vesicle dimensions using polycarbonate membrane extrusion techniques. Subsequently, these membrane sheets or vesicles are encapsulated onto the surface of nanoparticles (NPs) utilizing one of several methods: co-extrusion, ultrasonic treatment, or microfluidic electroporation. Additionally, the formation of membrane coatings on nanoparticles can occur spontaneously through electrostatic attraction between the charged particles of the nanoparticles and the membrane vesicles. This comprehensive approach ensures precise control over the size and surface characteristics of the CMC@NPs, catering to specific application needs across various biomedical fields (Table 1).

#### 3.2.1. Co-Extrusion

Co-extrusion technology assumes a pivotal role in the biomedical realm, especially in crafting biomimetic nanoparticles endowed with cell membrane functionalities. This innovative technique harnesses physical shear forces to blend and fuse cell membrane fragments with nanoparticles, yielding biomimetic nanoparticles imbued with specific biological attributes. Throughout the co-extrusion process, cell membrane vesicles and nanoparticles (NPs) undergo joint extrusion through polycarbonate membranes featuring progressively diminishing pore sizes. This procedure exerts robust forces capable of altering membrane structures, facilitating their reassembly on nanoparticle surfaces [31].

Nanoparticles generated via co-extrusion boast a uniform particle size distribution and excel in preserving membrane proteins’ integrity and biological functionalities. This preservation is pivotal for maintaining the biological activity inherent in the original cell membrane. Not only do these nanoparticles mirror natural cell surface properties, but they also enhance biocompatibility and targeting prowess, rendering them highly suited for crucial roles in drug delivery and disease diagnosis, where such attributes are indispensable.

The chief advantage of co-extrusion technology lies in its adeptness at systematically controlling both nanoparticle size and surface functionalization. This precision ensures the production of consistently high-quality, biologically active nanocarriers, stable and well-suited for clinical investigations. By leveraging physical forces for nanoparticle creation, co-extrusion circumvents potential biocompatibility concerns associated with chemical methods. This not only bolsters safety but also augments nanoparticle production efficiency, positioning co-extrusion as a dependable and efficient approach in nanotechnology applications.

#### 3.2.2. Ultrasonication

In contrast to the co-extrusion process, ultrasonication provides a simpler, faster, and more effective alternative for merging membrane vesicles with nanocarriers through acoustic wave technology. This method predominantly employs high-frequency acoustic waves to induce microbubble formation in the liquid. The rapid collapse of these bubbles generates powerful localized shear forces, facilitating the uniform distribution of cell membrane fragments across the nanoparticle surface. Consequently, this enhances the functionality and stability of the nanoparticles.

The propagation of sound waves in a liquid medium triggers various physical phenomenon that efficiently break down materials into very small particles within a short period, which is highly beneficial in the fine chemical and pharmaceutical industries. Specifically, the use of low-frequency ultrasound (20–100 kHz) enhances these effects, including particle size reduction, efficient mixing, mass transport, and the generation of intense shock waves associated with cavitation phenomena. Cavitation, characterized by the formation, growth, and implosion of high-energy microbubbles, serves as the primary mechanism by which ultrasonication reduces particle size in suspended materials in fluids, leading to significant structural changes [32]. Consequently, ultrasonication is regarded as a cost-effective and efficient alternative technique for optimizing the treatment and preparation of various materials.

Yokose et al. employed ultrasound-generated gas microbubbles to innovatively modify the oxidative state of the gas supply, enabling the selective production of solid and hollow particles. This method effectively transitions between oxidative and non-oxidative gas supplies by adjusting the ultrasound application, thereby controlling the particle formation process and the final structure of the particles [33].

In contrast, the study by Chang et al. investigated the impact of ultrasonic treatment on starch. Their findings revealed that ultrasonic treatment significantly reduced the viscosity of straight-chain starch solutions, facilitated the breakdown of straight-chain starch molecules, and resulted in a more concentrated molecular size distribution. These changes led to smaller, more uniform straight-chain starch nanoparticles (ANPs). Notably, the ultrasonic treatment preserved the crystal structure of the ANPs, maintaining their structural integrity [34]. This study underscores ultrasonic treatment as an efficient and cost-effective method for producing smaller, more homogeneous starch nanoparticles, optimized for industrial applications using nanoprecipitation techniques.

#### 3.2.3. Microfluidic Electroporation

Microfluidic electroporation is an innovative technique that integrates microfluidic and electroporation technologies. It involves applying an electric field to a microfluidic chip to temporarily disrupt the structure of both nanoparticles and cell membranes, facilitating the envelopment of nanoparticles by the cell membranes. Since its initial proof-of-concept demonstration in 2001, this method has gained substantial popularity among researchers due to its efficiency and precision in controlling the cell membrane wrapping process and enabling processing at the single-particle level.

A microfluidic chip specifically designed for electroporation not only enhances transfection efficiency but also lowers the voltage requirements. These chips are equipped with two inlets, allowing for the introduction and thorough mixing of nanoparticles and cell membranes within the channel. As this mixture flows through the electroporation zone, the electrical pulses generate micro-perforations in the cell membranes, assisting in the penetration of nanoparticles. Subsequently, the nanoparticles aggregate and are collected at the outlet as a mixture of CMC@NPs (cell membrane-coated nanoparticles). This technique demonstrates significant potential for precise biochemical manipulations at the microscale.

The efficacy of microfluidic electroporation in synthesizing cell membrane-coated nanoparticles (CM-NPs) was demonstrated in a study by Rao et al. In their experiments, they co-injected ferrite-containing magnetic nanoparticles (MNs) with vesicles derived from red blood cells (RBC-vesicles) into a microfluidic device. As these mixtures flowed through the electroporation region, they were subjected to electrical impulses that effectively facilitated the incorporation of the MNs into the RBC vesicles. Upon completion of this process, the research team successfully recovered MNs encapsulated in RBC membranes (RBC-MNs) from the device.

The team further evaluated the in vivo performance of these RBC-MNs by injecting them into experimental animals. Leveraging both the magnetic and photothermal properties of the MN cores and the ability of the RBC membrane shells to circulate in the bloodstream for extended periods, these nucleus–shell structured RBC-MNs have been utilized in advanced applications. They have shown promising results in enhanced magnetic resonance imaging (MRI) of tumors and photothermal therapy (PTT), indicating potential for clinical applications. This study underscores the versatility and precision of microfluidic electroporation in nanoparticle synthesis and its relevance in medical research and treatment modalities [35].

### 3.3. Bionic Cell Membrane Nanoparticles for Targeted Therapy in Acute Ischemic Stroke

#### 3.3.1. Biomimetic Nanoparticles for Red Blood Cell Membrane Functionalization

RBC is a type of cell in the blood, also known as a red blood cell or hemoglobin, whose main function is to carry oxygen and carbon dioxide for gas exchange in the body. The RBC membrane is the membrane at the periphery of RBCs, which encapsulates the cytoplasm of RBCs and is an important structure for maintaining the morphology and function of RBCs. Bionic nanoparticles formed by encapsulation of nanomedicines in the RBC membrane are a promising system for drug delivery. This system combines the superior drug delivery properties of nanoparticles with the biocompatibility, targeting, and stability of RBC membranes [36,37]. The outer layer of bionic nanoparticles is the RBC membrane, which is a natural component in the body, so it has good biocompatibility and is not easy to cause immune rejection. The outer layer of the RBC membrane can prolong its circulation time in the body, slow down the clearance of the drug, and improve the bioavailability and therapeutic effect of the drug. The proteins and receptors on the RBC membrane can afford the bionic nanoparticles the ability to target so that they can specifically recognize and bind to the target cells or tissues and achieve precise drug delivery. The RBC membrane wrapping can protect the internal drug from the interference of external factors and degradation, enhancing the stability of the drug and prolonging the effective period of the drug. The nature of bio-nanoparticles can be realized by modulating parameters such as the source of RBC membranes, the treatment method, and the drug loading mode, which has a certain degree of adjustability and flexibility (Table 2).

**Figure 2:**
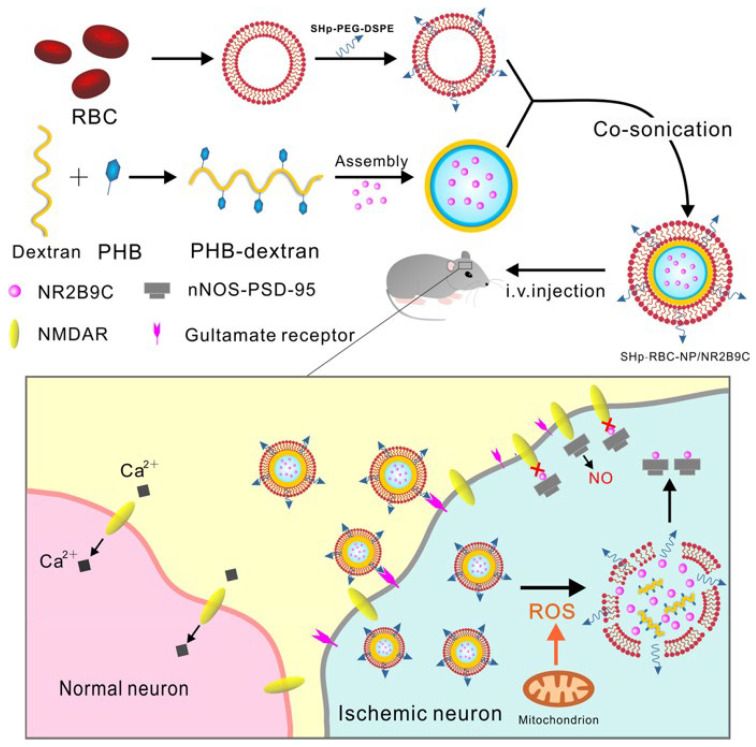
Schematic design of SHp-RBC-NP/NR2B9C. After intravenous injection, SHp-RBC-NP/NR2B9C could prolong the circulation life with its RBC-mimicking properties and then target the ischemic brain site via stroke-homing-peptide-mediated transcytosis [41]. Copyright 2018, American Chemical Society.

tPA is an important drug used to dissolve blood clots, especially in the treatment of thrombotic diseases such as IS. It restores blood flow by converting fibrinogen to fibrinolytic enzymes, which promotes thrombolysis. However, the effectiveness of tPA is limited by its short half-life, which means that it is present in the body for a short period and needs to be administered frequently to maintain the therapeutic effect. tPA has a half-life of only about 4–6 min, which means that it is cleared by the body very quickly after it is administered, limiting its prolonged action. To overcome the limitations of tPA’s short half-life, researchers are developing new therapeutic strategies, such as encapsulating tPA in a cell membrane bionic delivery system, which could allow it to acquire surface properties similar to those of natural cells, resulting in better compatibility with biological tissues in vivo and prolonging its presence in the circulatory system, increasing the drug’s bioavailability and reducing the frequency of dosing [38,39]. This technology is expected to improve the therapeutic efficacy of tPA, reduce its side effects, and bring new hope for the treatment of thrombotic diseases. Vankayala et al., to improve the bioavailability of the drug, designed a novel therapeutic nanoplatform system using a dual-stealth strategy [40]. The system consists of RBC-derived vesicles encapsulated with near-infrared fluorophore indocyanine green (ICG) and coupled to tPA on the surface. The dual-stealth strategy was adopted to overcome the limitations in drug delivery. RBC-derived vesicles have properties like those of natural red blood cells and can exist in the body for long periods without being easily removed. By encapsulating ICG on the vesicles, a photothermal conversion effect can be realized, which in turn promotes the thermotherapeutic effect in the treatment area. At the same time, coupling with tPA on the vesicle surface enables targeted delivery against thrombus. This nano platform system has the potential advantage of increasing the bioavailability of drugs and reducing the rate of drug clearance in the body. In addition to this, Lv et al. developed an RBC membrane-encapsulated polymeric nanoparticle for stroke-specific delivery of the neuroprotective agent NR2B9C against ischemic brain injury [41]. The nanocarrier consists of a ROS-responsive borate-modified dextran polymer core and an RBC membrane shell inserted with stroke homing peptide (SHp). Thus, these nanoparticles (SHp-RBC-NP) can target ischemic brain tissue and control the release of the neuroprotectant NR2B9C (Figure 2). The results of in vivo and in vitro experiments suggest that this nanocarrier has potential therapeutic effects on ischemic brain injury by reducing cytotoxicity and prolonging the somatic circulation of NR2B9C, thereby decreasing the extent of ischemic brain injury. This study provides new ideas for ischemic stroke therapy.

#### 3.3.2. Biomimetic Nanoparticles for Neutrophil Membrane Functionalization

Neutrophils play a vital role in the immune system, constituting a significant portion of peripheral blood, typically ranging from 50 to 70 percent of the total leukocyte count. Their importance is particularly notable in inflammatory conditions such as myocardial infarction. When tissue damage occurs in the myocardium, inflammatory signals prompt neutrophils to migrate toward the affected area, a process known as infiltration. Neutrophils primarily aid in the healing process by engulfing and eliminating pathogens and cellular debris from the injured tissue, while also releasing inflammatory mediators. Furthermore, neutrophils interact with endothelial cells, enhancing adhesion molecules to form adhesions at the site of inflammation. This facilitates the infiltration of other immune cells and fosters the inflammatory response, thereby enhancing immune cell aggregation in the affected area and improving the efficiency of the local immune response [42]. Neutrophil membranes possess natural biocompatibility and immune evasion properties, reducing recognition and clearance by the immune system [43]. Thus, encapsulating nanoparticles in neutrophil membranes prevents rapid removal from the circulatory system, prolonging their circulation time and enhancing therapeutic effectiveness. Additionally, neutrophil membranes can target damaged tissues, enabling precise drug release in affected areas, improving efficacy, and reducing side effects. This targeted drug delivery system is crucial for treating inflammatory diseases such as myocardial infarction. In summary, utilizing neutrophil membranes to encapsulate therapeutic nanoparticles shows promise in advancing disease treatment with innovative breakthroughs.

Fingolimod hydrochloride (FTY720), an anti-inflammatory agonist targeting the sphingosine 1-phosphate receptor, has undergone extensive research and has been approved for treating multiple sclerosis. Recent studies suggest its potential in the treatment of ischemic stroke as well [44,45]. While FTY720 exhibits notable neuroprotective and anti-inflammatory effects at high doses, its efficacy in treating ischemic stroke is constrained by the blood–brain barrier, thereby diminishing its effectiveness. Moreover, high doses have been associated with cardiovascular side effects, including bradycardia, atrioventricular obstruction, and reduced left ventricular function [46]. Furthermore, FTY720 may elevate the risk of infection due to its impact on the immune system. Therefore, while FTY720 shows promise in ischemic stroke therapy, its biocompatibility and safety require further study and evaluation. To facilitate the clinical application of FTY720 in ischemic stroke treatment, additional research and exploration of methods for precise dosing administration into affected brain regions are imperative. Such endeavors may optimize FTY720’s therapeutic efficacy while minimizing adverse reactions and side effects. Zhao et al. introduced an innovative therapeutic approach involving the development of ROS-responsive multi-premedicate nanoparticles (NRNs) coated with neutrophil membranes (NMs). These nanoparticles were engineered to transport FTY720 to ischemic brain tissues, to suppress the post-stroke inflammatory response and foster the recovery of central nervous system functions [47]. This study employed a sulfhydryl linker to conjugate amphiphilic drugs with FTY720 and fabricated these nanoparticles (NRNs) through self-assembly. The NRNs demonstrated targeted delivery to inflammatory brain lesions by leveraging inflammation-induced neutrophil recruitment, followed by the prompt release of FTY720 in the oxidative microenvironment of the ischemic brain. This targeted release facilitated efficient FTY720 delivery to brain tissues, prompting the selective polarization of microglia from the pro-inflammatory M1-type to the anti-inflammatory M2-type. Consequently, damaged neurons were notably rescued, and neurological recovery following ischemic stroke was promoted. Moreover, the study revealed that in vivo administration of NRNs exhibited minimal cardiotoxicity or infection risk compared to intravenous injection of FTY720 alone. This underscores the significantly enhanced biocompatibility of the multi-drug nanoplatforms, further supporting their potential for clinical applications.

**Figure 3:**
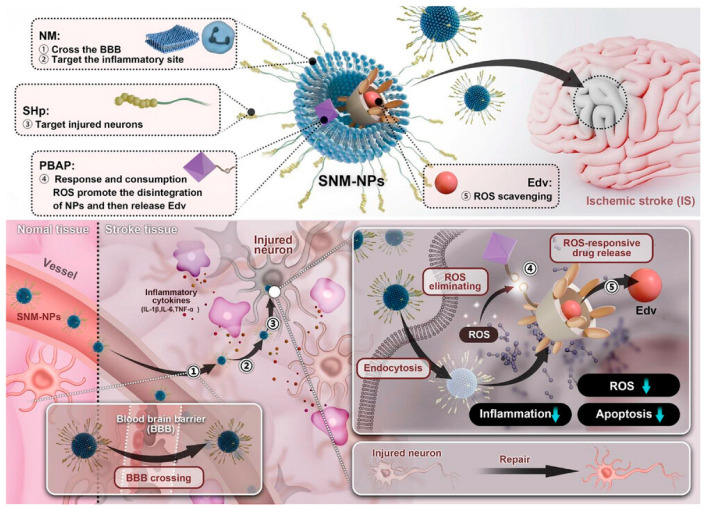
Composition and stepwise targeting mechanism of SNM-NPs and their therapeutic effect on CIRI [48]. Copyright 2023, Wiley-VCH GmbH.

In another study, Dong et al. achieved a significant milestone by constructing a precise biomimetic drug delivery system, termed SNM-NPs, with stepwise targeting capabilities (Figure 3). This innovative nano platform holds promise for treating ischemic stroke [48]. Edaravone (Edv), a pivotal drug in stroke therapy renowned for its ROS scavenging abilities, faces challenges such as poor blood–brain barrier permeability, short half-life, and potential toxicity at high doses. SNM-NPs effectively addressed these limitations by efficiently delivering Edv to the site of cerebral ischemia-reperfusion injury in rats. This success can be attributed to the NM encapsulation and SHp modification of SNM-NPs, which facilitated blood–brain barrier penetration, inflammation, ROS mitigation, and precise targeting of injured neurons at the CIRI site. Firstly, NM encapsulation enabled SNM-NPs to traverse the blood–brain barrier and achieve targeted delivery to inflamed regions. Secondly, SHp modification enhanced the specificity of SNM-NPs towards damaged neurons, significantly boosting their targeting efficiency. Moreover, SNM-NPs adopted a stepwise targeting strategy, resulting in reduced in vivo drug concentrations and inhibition of neuroinflammation by scavenging excess ROS. This ROS scavenging mechanism not only upregulated Bcl2 expression, inhibited Bax function, and suppressed Caspase 3 activation but also hindered neuronal apoptosis and microtubule repair. Initial experiments confirmed the favorable safety profile of SNM-NPs in intravenous treatment and in vitro cellular assays, laying a robust foundation for their further development as effective and safe therapeutics for CIRI and related conditions. Furthermore, the proposed stepwise targeting and precise drug delivery approach holds promise for the precision treatment of other brain disorders associated with local oxidative stress and inflammation.

#### 3.3.3. Biomimetic Nanoparticles for Platelet Membrane Functionalization

Platelets play an important role in the treatment of ischemic stroke, not only participating in thrombosis and inflammation regulation but also having the function of promoting vascular repair [49]. First, in ischemic stroke, vascular obstruction leads to localized thrombosis, which results in inadequate blood supply to the brain. Platelets are involved in the process of thrombosis, promoting thrombosis through aggregation and activation. In treatment, antiplatelet drugs can be utilized to inhibit platelet activity and reduce the risk of thrombosis, thereby helping to restore blood supply to the brain. Second, ischemic stroke induces a local inflammatory response, and platelets can release a variety of inflammatory mediators, such as platelet factor, to participate in the regulation of inflammation. In treatment, the inflammatory response can be reduced by regulating the activity of platelets to reduce the damage to brain tissue. Finally, ischemic stroke damages the vascular structure of the brain, and platelets can release bioactive substances such as growth factors to promote the repair and regeneration of blood vessels. In treatment, this repair function of platelets can be utilized to accelerate the recovery of blood vessels in the brain and improve blood circulation. Therefore, the regulation and intervention for platelets can be one of the important strategies for the treatment of ischemic stroke. Studies have shown that platelet aggregation has been applied in clinical practice. Saccaro et al. studied 28 patients with AIS and 4 patients with transient ischemic attack (TIA), compared with 29 controls [50]. The study collected blood and urine samples from the patients at various time points after onset (4.5 h, 24 h, and 3 months after D0) and once from the controls at D0. It was found that, at D0, patients exhibited a significant decrease in platelet 5-hydroxytryptamine transporter (SERT) density (*p* = 10^−5^), a significant increase in platelet 5-HT2A receptor (5-HT2AR) density (*p* < 10^−6^), a significant increase in the plasma tryptophan/kynurenine ratio (K/T) (*p* = 10^−5^), and a significant increase in urinary 5-HT (*p* = 0.011), and 5-hydroxyindoleacetic acid (5-HIAA) levels were significantly increased (*p* = 0.003). The study concludes that, for the first time, a hyperacute dysregulation of the serotonergic axis, as well as hyperacute and persistent activation of the tryptophan–kynurenine pathway in patients with cerebral ischemia, has been observed. Meanwhile, L-tryptophan (TRP) plays an important role in the regulation of homeostasis, immunity, and neuronal function [51]. Metabolic changes in TRP are involved in the pathophysiological processes of a variety of CNS diseases. TRP is metabolized through two main pathways: the kynurenine pathway and the methoxy-indole pathway. In the kynurenine pathway, TRP is first metabolized to kynurenine, then quinolinic acid, o-aminobenzoic acid, 3-hydroxy kynurenine, and finally 3-hydroxy o-aminobenzoic acid, in that order. In contrast, in the methoxy indole pathway, TRP is metabolized to 5-hydroxytryptamine and melatonin.

Platelet mimetic nanoparticles are usually polymeric nanoparticles coated by platelet membranes with surface properties like those of natural platelets, thus mimicking the adhesion and targeting ability of platelets. Platelet-mimetic nanoparticles show promise as a targeted drug delivery system for treating thrombotic diseases, offering enhanced drug targeting, improved therapeutic outcomes, and reduced side effects. Quan et al. introduced a groundbreaking tPA delivery platform (APLT-PA), leveraging platelet membrane and the membrane-associated protein V to augment tPA’s efficacy at thrombosis sites. The targeting effect of tPA was enhanced, thus improving its therapeutic effect on acute ischemic stroke. Membrane link protein V inhibits thrombosis by impairing phosphatidylcholine (PS), an effect that can be eliminated by phosphatidylcholine micelles. PS is a phospholipid found in platelets and other cell membranes, which plays an important role in thrombosis. By interacting with PS, membrane-bound protein V can prevent platelet aggregation and thrombosis. However, phosphatidylcholine micelles can bind to membrane-bound protein V and neutralize its effect, thereby attenuating its inhibitory effect on thrombosis. From in vitro and in vivo experiments, APLT showcased remarkable efficacy in targeting activated platelets by combining with PS and activated platelet membrane proteins, resulting in robust adhesion to activated platelets in vitro and thrombotic sites in vivo. In a murine model of photochemically induced AIS, a single dose of APLT-PA exhibited significant thrombolytic effects and notably improved neurological function within just 7 days. This research introduces a novel and safe biomimetic platelet nanodrug for precise therapeutic thrombolysis in AIS, providing a novel theoretical framework for the design and development of biomimetic cellular nanodrugs.

**Figure 4:**
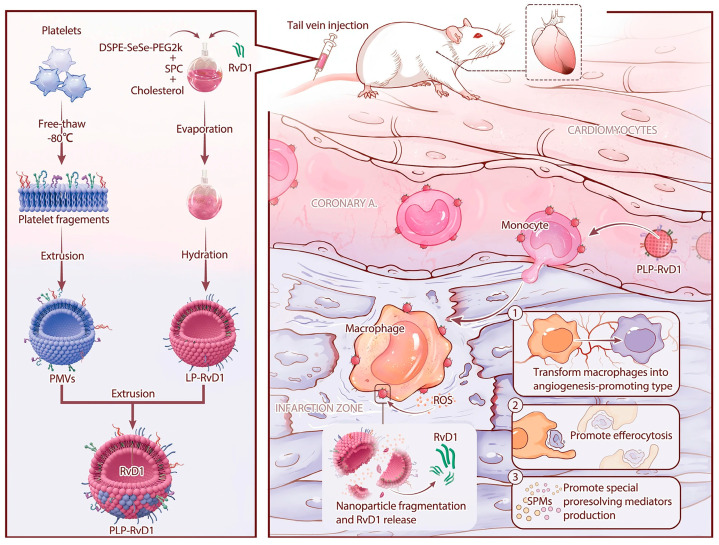
PLP-RvD1 fabrication and targeted treatment for myocardial ischemia-reperfusion injury [51]. Copyright 2022, *Journal of Nanobiotechnology*.

In another study, Weng et al. designed a platelet-mimetic Resolvin D1 (RvD1) delivery platform that could act in a targeted manner on the injured area to promote dead cell clearance, SPM generation, and angiogenesis during the repair phase (Figure 4) [22]. RvD1 plays a facilitating role in macrophage differentiation toward a pro-angiogenic phenotype, thereby contributing to the repair of injured tissues [52]. This affords RvD1 the potential to be a small molecule drug that targets macrophages and can be used to promote repair after myocardial injury. In addition, RvD1 has been reported to offer effective protection to several critical organs, including the heart, brain, and kidneys, against ischemia-reperfusion injury [53,54,55]. These findings serve as a crucial theoretical and empirical foundation for utilizing RvD1 in the treatment not only of myocardial injury but also of injuries affecting other organs. This platelet-mimetic RvD1 delivery platform is ROS-responsive, facilitating localized and controlled release by harnessing circulating chemotactic monocytes for targeted delivery of RvD1 to the injury site. Through in vivo experiments, this platform effectively concentrates RvD1 at the injury site, facilitating clearance of dead cells, production of specialized pro-resolving mediators (SPMs), and angiogenesis, ultimately leading to substantial enhancement in cardiac function. By integrating drug bio-protection, targeted delivery, and controlled release, this delivery system holds immense promise for clinical translation. In addition, Cui et al. designed a bionic nanoparticle (PM-GB) of ginkgolide B (GB) encapsulated by platelet membranes for the treatment of ischemic stroke [56]. GB is an active ingredient extracted from the leaves of Ginkgo biloba, and its main components are flavonoids. This compound has been extensively studied and has been suggested to have a variety of pharmacological effects including anti-inflammatory, antioxidant, neuroprotective, and Vaso-protective [57,58,59] effects. In AIS, the inflammatory response is one of the important pathological processes. The occurrence and development of inflammation lead to further damage to brain tissue. GB has the effect of inhibiting the inflammatory response, which can reduce the inflammatory damage of brain tissue and help to protect the nerve cells. IS leads to hypoxia and ischemia of brain tissue, which generates a large number of free radicals and causes oxidative stress, resulting in lipid peroxidation of cell membranes and cellular damage. GB has an antioxidant effect, which neutralizes the free radicals and reduces the damage caused by oxidative stress to brain tissue. In addition, iron ions play an important role in ischemic stroke, which can induce cellular iron death and aggravate brain tissue damage. GB is claimed to have the effect of inhibiting iron death, which can help to reduce brain damage. From the experimental results, PM-GB attenuated iron uplift and inflammation in rats with a middle cerebral artery occlusion model by inhibiting oxidative stress, which in turn protected nerve cells and promoted motor recovery. These results provide strong experimental support for the potential efficacy of PM-GB in the treatment of IS.

#### 3.3.4. Biomimetic Nanoparticles for Exosome Membrane Functionalization

Exosomes, being extracellular vesicles laden with lipids, nucleic acids, proteins, and bioactive molecules, exhibit diameters ranging from 30 nm to 150 nm. With their inherent immunogenicity, biocompatibility, and homing prowess, they serve as pivotal tools in immunomodulation and drug conveyance. Of particular interest is their ability to traverse the blood–brain barrier, precisely targeting cerebral regions, thus facilitating drug accumulation within brain lesions. Consequently, leveraging bio-nanoparticles coated with exosome membranes holds significant promise in stroke therapy [60].

He Li and colleagues [61] conducted an investigation into the role of exosomes in the protective mechanism of cerebral ischemic preconditioning (cerebral-IPC) against cerebral ischemia/reperfusion (I/R) injury. Their findings demonstrated a significant reduction in cerebral ischemia and reperfusion injury, thereby indicating a neuroprotective effect. Through miRNA microarray analysis, they identified differentially expressed miRNAs within exosomes derived from sham-operated (S-exosomes) and ischemic preconditioned (IPC-exosomes) mice, subsequently elucidating their target genes through database interrogation. In vitro experiments involved the treatment of both control and oxygen–glucose deprivation/reoxygenation (OGD/R) cells with IPC exosomes, miRNA mimics, or inhibitors targeting specific proteins. Viability, oxidation levels, stress responses, and apoptosis rates were assessed to evaluate cellular responses. Additionally, pathway activation was delineated by quantifying pertinent protein levels. Notably, miR-451a, which targets Rac1, exhibited upregulation in IPC exosomes compared to S-exosomes. Further experimentation utilizing miR-451a mimics and the Rac1 inhibitor NSC23766 reversed the OGD/R-induced activation of Rac1 and its downstream pathways. Yong Wang and colleagues [62] devised an exosome-based therapeutic approach, termed anti-CHAC1 ADSC-Exo, targeting iron death in cerebral ischemia/reperfusion injury. Through bioinformatics analysis, they identified the CHAC1 gene as a key contributor to iron death in ischemic stroke. Notably, miR-760-3p was found to downregulate CHAC1 expression and potentially be enriched within ADSC-Exo. Following the successful isolation of ADSC-Exo, immunofluorescence techniques demonstrated their efficient delivery to the brain via intranasal (IN) administration. Consequently, this approach effectively ameliorated neurobehavioral deficits and attenuated iron death in mice. Importantly, miR-760-3p encapsulated within ADSC-Exo exerted its therapeutic effect by targeting CHAC1 specifically within neurons. Chao Yang and colleagues [63] investigated the utilization of nanocapsules for delivering antibodies to brain tumors. Their research demonstrated that exosomes from primary glioma cells increased LCN2 protein levels through the JAK-STAT3 pathway. LCN2, in turn, enhanced endothelial cell membrane fluidity, aiding the penetration of MPC nanocapsules through the blood–brain barrier (BBB) both in vitro and in vivo. Inhibiting exosome release decreased MPC nanocapsule transport across the BBB while pre-administering tAb nanocapsules before blocking exosome release improved therapeutic outcomes in patient-derived glioma xenografts. Additionally, the study identified MK2206 as an AKT inhibitor that effectively suppresses exosome production in gliomas. Notably, pre-treatment with tAb nanocapsules enhanced the efficacy of combined treatment with nanocapsules and MK2206. In conclusion, the study elucidated the role of LCN2 in exosome-mediated BBB penetration in gliomas, thereby facilitating the delivery of nanocapsules.

Exosomes derived from astrocytes, known as astrocyte-derived exosomes (ADEXs), have emerged as promising vehicles for intercellular signaling and are implicated in various diseases, including Alzheimer’s disease, hypoxic-ischemic brain injury, traumatic brain injury, Parkinson’s disease, spinal cord injury, and ischemic stroke. In their study, Yang Deng et al. [64] examined the role and mechanism of exosomes in an AIS model, both in vivo and in vitro, focusing on astrocyte-mediated neuroprotection. They identified Nampt as a crucial regulator of autophagy, with its expression notably elevated in oxygen–glucose deprivation/reperfusion (OGD/R)-ADEXs. Knockdown of Nampt in astrocytes attenuated the protective effects observed with OGD/R-ADEXs. Mechanistically, Nampt promoted autophagy and mitigated cell death by modulating the AMPK/mTOR signaling pathway, recognized as pivotal in autophagy regulation following AIS. The release of Nampt from OGD/R-ADEXs demonstrated neuroprotective effects during acute ischemic stroke-induced neuronal injury by targeting the AMPK/mTOR signaling pathway to induce autophagy. This study unveils a novel key factor in exosome secretion by OGD/R-treated astrocytes, shedding light on its role in regulating autophagy and eliciting neuroprotection in a mouse stroke model.

#### 3.3.5. Macrophage Membrane-Functionalized Biomimetic Nanoparticles

Cell membrane-modified nanodrugs exhibit dual properties of nano-formulation and biofilm characteristics. Encapsulated within the cell membrane carrier, these drugs can traverse the BBB more effectively, thereby enhancing their brain-targeting efficacy [65]. Macrophages, integral to innate immunity and widely distributed in body tissues, play a pivotal role in the monocyte macrophage system, predominantly governed by microglia in the brain. Monocyte–macrophages possess the capability to alter their morphology and breach the BBB through blood cell exudation, leveraging their inherent ability to cross this barrier. Consequently, drugs engineered with macrophage membrane modifications hold promise for improved efficacy in encephalopathy treatment [66]. Furthermore, during the acute inflammatory phase, monocytes/macrophages exhibit sustained migration toward the ischemic region of the brain [67].

Tianshu Liu et al. [68] developed an innovative delivery system for treating ischemic stroke via nasal administration. Their strategy involved developing a liposomal drug delivery system simultaneously transporting panax notoginseng total saponins (PNSs), and ginsenoside Rg3 (Rg3). To enhance targeted delivery to ischemic brain regions, they integrated macrophage membranes onto the liposome surface. Upon administering MM-Lip-Rg3/PNS for 14 days, middle cerebral artery occlusion (MCAO) rats demonstrated notable enhancements in survival rates, body weight, and neurological function scores. Furthermore, reductions in neuronal apoptosis and inflammatory cell infiltration were evident, contributing to the preservation of ischemic brain tissue integrity. In vivo animal experiments validated that MM-Lip-Rg3/PNS treatment mitigated apoptosis, inflammatory responses, and oxidative stress by modulating various targets (including AKT1, VEGFA, EGFR, CASP3, STAT3, and MAPK1) and pathways (including PI3K/Rap1/cAMP/IL-17/TNF signaling pathways), thus presenting a promising therapeutic avenue for ischemic stroke. Yu Long et al. [69] investigated the modification of macrophage membranes on the surface of baicalein liposomes (BA-LP) and observed a significant enhancement in the targeting efficiency of the modified baicalein liposomes (MM-BA-LP) compared to unmodified baicalein liposomes (BA-LP). Employing a pharmacodynamic study utilizing a rat middle cerebral artery embolization (MCAO) model, they evaluated the therapeutic efficacy of MM-BA-LP on cerebral ischemia-reperfusion injury (CIRI). The findings indicated that MM-BA-LP markedly ameliorated neurological deficits, reduced infarct volume, and improved brain histopathological status in MCAO rats compared to BA-LP. Moreover, MM-BA-LP exhibited enhanced cerebral targeting of baicalein, improved blood circulation, and superior neuroprotective effects over BA-LP in the MCAO rat model.

The inherent characteristics of macrophage membranes, such as immune evasion and resistance to clearance, contribute to enhancing the stability and bioavailability of nanoparticles in vivo. Utilizing macrophage membranes to encapsulate nanoparticles can thus improve their targeting specificity and biological efficacy, presenting a promising strategy for enhancing drug delivery and therapeutic outcomes.

#### 3.3.6. Biomimetic Nanoparticles for Neural Stem Cell Membrane Functionalization

Stem cells possess advantageous traits including self-renewal capacity, pluripotent differentiation potential, and low immunogenicity. Neural stem cells (NSCs) have emerged as a promising avenue in the treatment of ischemic stroke (IS) due to their innate propensity to migrate towards ischemic brain regions. NSCs not only exhibit the capability to traverse the blood–brain barrier but also demonstrate remarkable targeting specificity towards ischemic brain territories [70].

**Figure 5:**
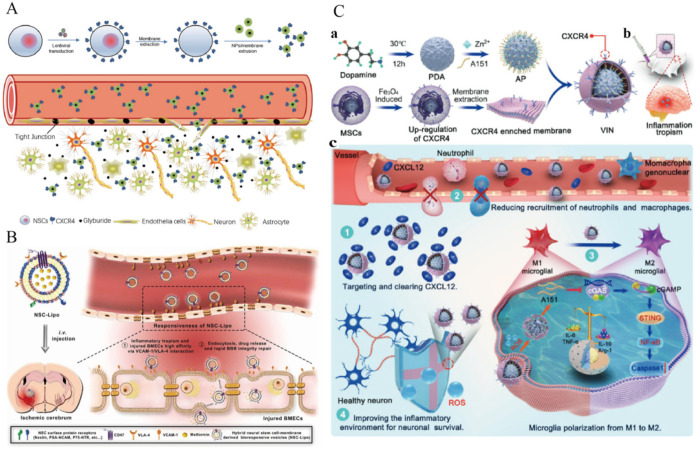
(**A**) To deliver therapeutic agents to ischemic brain regions, researchers coated PLGA nanoparticles (NPs) with membranes from CXCR4-overexpressing neural stem cells (NSCs). They first genetically modified NSCs to enhance CXCR4 expression using lentiviral transduction. The extracted NSC membranes were then applied to the PLGA NPs via extrusion. Upon intravenous administration, these engineered composite nanoparticles (CMNPs) selectively accumulated in ischemic areas due to the interaction between CXCR4 on the membrane and its ligand, SDF-1, which is abundant in ischemic microenvironments. Once localized, CMNPs released therapeutic agents, like glyburide, aiding in stroke repair and recovery [69]. Copyright 2019, WILEY-VCH Verlag GmbH & Co. KGaA, Weinheim. (**B**) After injection, VLA-4 on neural stem cell membranes migrated to bioreactive vesicles (NSC-Lipo), enabling natural inflammatory targeting of injured brain microvascular endothelial cells (BMECs) through VLA-4/VCAM-1 interaction. Subsequently, NSC-Lipo facilitated targeted delivery of anti-inflammatory drugs, suppressing the inflammatory response in injured BMECs and rapidly restoring blood–brain barrier integrity. However, it is crucial to acknowledge that inhibiting VCAM-1 expression disables the targeting potential of these bioresponsive vesicles [70]. 2023 Elsevier Ltd. All rights reserved. (**C**) Illustrative depiction of constructing a multifunctional immunosuppressive nanoparticle incorporating a CXCL12 mimetic decoy for treating ischemic stroke, aiming to modulate the hyperactive immune environment in the brain. (**a**) Illustrative representation of the steps involved in creating the Versatile Immunosuppressive Nanoparticle (VIN). (**b**) Following intravenous administration, the VIN enriched with CXCR4 exhibits a targeted response to inflammation in ischemic brain regions. (**c**) The stages of action include the following: (1) Designing membrane vesicles that overexpress CXCR4 to act as a nanoscale decoy, neutralizing the pro-inflammatory molecule CXCL12. (2) VIN disrupts the interaction between CXCR4 and CXCL12, diminishing neutrophil and monocyte infiltration. (3) Reactive oxygen species (ROS) produced by A151 block the cGAS–STING pathway in microglia. (4) VIN promotes an anti-inflammatory response by converting microglia to an M2-like phenotype and scavenges ROS, thereby shielding neuronal cells from oxidative stress-induced apoptosis [72]. Copyright 2021, Wiley-VCH GmbH.

Junning Ma and colleagues [71] explored the utilization of neural stem cell (NSC) membranes encapsulated onto the surface of poly(lactic-co-glycolic acid) nanoparticles (NPs) for targeted drug delivery to ischemic brain regions. They designated the NSC membrane-coated NPs as MNP and those coated with NSCs overexpressing CXCR4 as CMNP. Their study revealed that encapsulation with NSC membranes significantly enhanced NP accumulation in ischemic brain tissues, with further improvement in delivery efficiency observed when utilizing membranes from NSCs engineered to overexpress CXCR4. Additionally, CMNPs markedly augmented the therapeutic efficacy of glibenclamide in treating brain injuries. These findings suggest the potential translation of glibenclamide-loaded CMNPs into clinical applications for managing human brain injuries (Figure 5A). Honghui Wu and collaborators [72] conducted a study investigating the utilization of recombinant neural stem cell (NSC) membranes and conventional liposomes to create bioreactive vesicles (NSC-Lipo). They found that VLA-4 expressed on NSC membranes exhibited superior efficacy in managing human brain injury patients compared to NSCLipo. This enhanced effectiveness was attributed to the specific recognition and binding of VLA-4 on NSC membranes to VCAM-1 on damaged brain microvascular endothelial cells (BMECs) via VCAM-1/VLA-4 interactions. Subsequently, metformin was loaded into these vesicles as an anti-inflammatory agent. Following a single systemic administration in mice with ischemic stroke, NSC-Lipo loaded with metformin demonstrated the ability to recognize and target damaged BMECs, leading to endocytosis in the lesion area. Consequently, the released metformin mitigated inflammation in the BMECs, facilitated rapid repair of the blood–brain barrier (BBB) integrity, and initiated neuroprotective mechanisms, ultimately resulting in improved survival rates in mice (Figure 5B).

#### 3.3.7. Biomimetic Nanoparticles Functionalized with Engineered Cell Membranes

Cell membranes, being natural biological constituents, confer excellent compatibility upon the nanoparticles they encase, thereby mitigating the risk of immune rejection [73]. Tailored modifications of engineered cell membranes allow for the incorporation of specific targeting molecules, facilitating precise localization of nanoparticles to target tissues or cells and thereby enhancing therapeutic efficacy [74]. Moreover, these membranes can be personalized to accommodate diverse therapeutic requirements, including the addition of specific receptors, antibodies, or other bioactive moieties to augment targeting and therapeutic potential [75]. These inherent advantages position engineered cell membrane-encapsulated nanoparticles as a highly promising avenue in drug delivery, particularly within the realms of precision medicine and targeted therapy.

Jinjin Shi et al. [76] developed multifunctional immunosuppressive nanoparticles, termed ionic decoy-integrated VINs, designed for managing an overactive brain immune microenvironment (Figure 5C). The VINs (Vesicles Integrated with Nanoparticles) were created by enclosing A151-loaded polydopamine nanospheres (PDAs) within mesenchymal stem cell (MSC) membrane vesicles enriched in CXCR4. These engineered MSC vesicles not only improved nanoparticle migration to the damaged brain area and efficiently targeted the CXCR4–CXCL12 axis but also acted as a “nano decoy” to sequester the inflammatory mediator CXCL12. This action prevented the infiltration of peripheral inflammatory cells such as neutrophils and monocytes. A151, an oligonucleotide containing telomerase repeats, inhibited the cGAS–STING pathway in microglia, leading to their polarization towards an anti-inflammatory M2-like state. The effective loading of A151 onto the safe PDA core of VINs was facilitated through a Zn^2+^-mediated bridge. In the high-ROS (Reactive Oxygen Species) environment typical of inflammation sites, PDAs oxidized, causing the dissociation of Zn^2+^ and subsequent A151 release. This mechanism ensured precise drug delivery and controlled release within the brain. Moreover, PDAs exhibited efficient ROS scavenging properties, protecting neuronal cells from ROS-induced apoptosis in the inflammatory environment. Overall, this study demonstrated VINs as a comprehensive therapeutic strategy, combining precise inflammation targeting, controlled drug release, ROS scavenging, and dual anti-inflammatory effects. This approach showed significant efficacy in improving ischemic rat survival rates, reducing infarct volume, and preserving post-stroke neurons.

Li Luo et al. [77] synthesized the ROS-responsive amphiphilic copolymer HBA-OC-PEG2000 (HOP) by chemically polymerizing 4-hydroxybenzaldehyde (HBA) with oxalyl chloride (OC) and polyethylene glycol 2000 (PEG2000). Rapamycin (RAPA) was loaded into HOP nanoparticles (NPs), which were then coated with bioengineered cell membranes overexpressing CXCR4 to form RAPA@BMHOP. This approach not only facilitated the targeted delivery of RAPA@BMHOP, exploiting CXCR4/SDF-1-dependent signaling for efficient accumulation at sites overexpressing CXCR4 and involved in SDF-1-mediated recognition, but also enabled selective control of cargo release via ROS-responsive degradation. RAPA@BMHOP played a pivotal role in regulating cellular ROS dynamics, thereby mitigating oxidative damage, neuroinflammation, and subsequent injury in the affected region. Moreover, the favorable pharmacokinetic profile, stealthy effect, and good biocompatibility of RAPA@BMHOP were attributed to the cell membrane coating technology. Endocytosis and internalization of RAPA@BMHOP by receptor cells were mediated by the CXCR4/SDF-1 mechanism, facilitating targeted delivery of therapeutic agents to intracellular sites with excessive ROS. Cleavage of oxalate bonds by HBA-OC-PEG2000 led to the release of RAPA into the microenvironment. Consequently, RAPA@BMHOP enabled active delivery to ischemic regions, thereby enhancing the therapeutic efficacy against I/R injury.

## 4. Conclusions and Outlook

Biomimetic nanoparticles functionalized with cell membranes present an innovative approach for treating acute ischemic stroke. These nanoparticles enhance drug delivery efficiency and targeting by mimicking the behavior and structure of natural cells. In ischemic stroke models, this nanosystem has shown an excellent capacity to penetrate the blood–brain barrier and directly deliver therapeutic agents to damaged brain cells. Moreover, the cell membrane coating on these nanoparticles effectively camouflages them, reducing immune clearance and prolonging the drug’s circulation time in the bloodstream, thereby minimizing potential immunogenic reactions.

Specifically, the functionalization of the cell membrane not only enhances the solubility and stability of drug molecules but also optimizes the dynamics of drug release, facilitating more precise and controlled treatment. Studies have demonstrated that nanoparticles utilizing this technology are more effective in mitigating the neuroinflammatory response following brain injury and in promoting the repair and regeneration of damaged neural tissue. Consequently, cell membrane-functionalized bio-nanoparticles not only augment the direct efficacy of treatment but also contribute to reducing the severity of post-stroke sequelae, enhancing patient recovery quality. In conclusion, biomimetic nanoparticles functionalized with cell membranes show considerable potential in the treatment of acute ischemic stroke. By offering a therapeutic pathway that is both effective and safe, this emerging technology is poised to become a vital tool in future stroke treatment strategies. Although biomimetic cell membrane nanoparticles have demonstrated potential in drug delivery and therapeutics, they still face multifaceted technical, safety, and regulatory challenges in clinical translation. Interdisciplinary collaboration and sustained research investment are needed to overcome these limitations and ultimately realize their application in clinical medicine.

Although significant progress has been made in experimental studies, biomimetic nanoparticles face several challenges in the treatment of ischemic stroke. These include biocompatibility and safety, the kinetics of drug release and control, and effective translation from the laboratory stage to clinical practice. Biocompatibility is crucial, particularly in ensuring that nanoparticles do not trigger immune responses or cause long-term toxic effects during extended treatment periods. Moreover, effective drug release and control mechanisms are essential for nanoparticles to serve as drug carriers, necessitating the development of systems capable of releasing drugs at specific times and locations to achieve optimal therapeutic efficacy. While laboratory studies have demonstrated the potential of nanoparticles in stroke treatment, several challenges remain for their effective clinical application. These challenges include the requirement for large-scale production, rigorous adherence to clinical trial standards, and the need for long-term assessment of therapeutic efficacy. Future research should prioritize optimizing nanoparticle design, encompassing size, shape, and surface properties, to enhance their ability to penetrate the blood–brain barrier and improve targeting within the brain. Additionally, the advancement of multifunctional nanoparticle systems that integrate diagnostic and therapeutic functionalities is anticipated to offer more precise and effective treatment options for stroke patients. Addressing these challenges demands close interdisciplinary collaboration among experts from diverse fields such as biomedical engineering, nanotechnology, neuroscience, and pharmacology.

Limitation: Despite significant progress in experimental studies, cell membrane-functionalized biomimetic nanoparticles still confront numerous challenges in clinical applications. Future research should concentrate on optimizing the design of nanoparticles, including their size, shape, and surface properties, to enhance their ability to penetrate the blood–brain barrier and improve targeting within the brain. It is also crucial to systematically evaluate the biodistribution, metabolism, and long-term safety of these nanosystems. Additionally, the development of multifunctional nanoparticle systems that integrate diagnostic and therapeutic functions may offer more precise and effective treatment options for stroke patients.

With the rapid advances in nanotechnology and cell engineering, we anticipate significant breakthroughs in the use of this technology in the treatment of acute ischemic stroke.

## Figures and Tables

**Table 1:** Methods of cell membrane preparation.

Methods	Principle	Advantages
Co-extrusion	Physical shear-dependent mixing and fusion of cell membrane fragments with nanoparticles to generate biomimetic nanoparticles with specific biological functions	Ability to systematically control nanoparticle size and surface functionalization, resulting in highly consistent and bioactively stable nanocarriers for clinical studies
Ultrasonication	The use of high-frequency sound waves to form tiny bubbles and generate strong localized shear forces when they collapse violently helps to uniformly cover cell membrane fragments on the surface of nanoparticles	Not only is it simpler and faster, but it also effectively promotes the fusion of membrane vesicles with nanocarriers via acoustic wave technology
Microfluidic electroporation	Temporarily perturbing the structure of nanoparticles and cell membranes using an electric field applied to a microfluidic chip, thereby enabling the cell membrane to wrap around the nanoparticles	Efficient and precise control of cell membrane encapsulation with single-particle level processing

**Table 2:** Summarizing the characteristics of bionic cell membrane nanoparticles for stroke applications.

Type	Advantages	Disadvantages	Limitations of Clinical Translation
Red blood cell membrane functionalization	Prolong its circulation time in the body, slow down the clearance of the drug, and improve the bioavailability and therapeutic effect of the drug, and they can specifically recognize and bind to the target cells or tissues, and achieve precise drug delivery	Recurrence and regulation of function; complex and costly; requires special storage conditions to maintain its functionality and performance	Complex preparation and standardization; stability and long-term storage; Targeted capacity and therapeutic effects; the challenge of clinical trials
Neutrophil membrane functionalization	Biocompatibility and immune evasion properties, reducing recognition and clearance by the immune system	Recurrence and regulation of function; scaled production and application limitations	Complex preparation and standardization; stability and long-term storage; targeted capacity and therapeutic effects; the challenge of clinical trials
Platelet membrane functionalization	Enhanced drug targeting, improved therapeutic outcomes, and reduced side effects	Recurrence and regulation of function; scaled production and application limitations; drug loading and release efficiency	Stability and long-term storage; targeted capacity and therapeutic effects; the challenge of clinical trials
Exosome membrane functionalization	With their inherent immunogenicity, biocompatibility, and homing prowess, they serve as pivotal tools in immunomodulation and drug conveyance	Recurrence and regulation of function; drug loading and release efficiency; scaled production and application limitations	Complex preparation and standardization; stability and long-term storage; targeted capacity and therapeutic effects; the challenge of clinical trials
Macrophage membrane functionalization	Traverse the blood–brain barrier (BBB) more effectively, thereby enhancing their brain-targeting efficacy	Recurrence and regulation of function; drug loading and release efficiency; scaled production and application limitations	Stability and long-term storage; targeted capacity and therapeutic effects; the challenge of clinical trials
Neural stem cell membrane functionalization	Traits including self-renewal capacity, pluripotent differentiation potential, and low immunogenicity	Recurrence and regulation of function; drug loading and release efficiency; scaled production and application limitations	Complex preparation and standardization; stability and long-term storage; targeted capacity and therapeutic effects; the challenge of clinical trials
Engineered cell membranes functionalization	Allow for the incorporation of specific targeting molecules, facilitating precise localization of nanoparticles to target tissues or cells and thereby enhancing therapeutic efficacy	Recurrence and regulation of function; drug loading and release efficiency; scaled production and application limitations	Complex preparation and standardization; stability and long-term storage; targeted capacity and therapeutic effects; the challenge of clinical trials

## Abbreviations

Abridgement	Generic Term
ADEXs	astrocyte-derived exosomes
AIS	acute ischemic stroke
ANPs	amylose starch nanoparticles
ATP	adenosine triphosphate
BA-LP	baicalein liposomes
BBB	Blood–brain barrier
BMECs	brain microvascular endothelial cells
CIRI	cerebral ischemia and reperfusion injury
CMC@NPs	cell membrane-coated nanoparticles
Edv	edaravone
FTY720	fingolimod hydrochloride
GB	ginkgolide B
GBD	global burden of disease
H^+^	hydrogen ion
HBA	4-hydroxybenzaldehyde
HT	hemorrhagic transformation
ICG	indocyanine green
IL-1	interleukin-1
IL-1β	interleukin-1β
IL-6	interleukin-6
IL-10	interleukin-10
I/R	ischemia/reperfusion
IS	ischemic stroke
MCAO	middle cerebral artery occlusion
MM-BA-LP	modified baicalein liposomes
MMPs	matrix metalloproteinases
MNs	magnetic nanoparticles
MRI	magnetic resonance imaging
MSC	mesenchymal stem cell
NMs	neutrophil membranes
NO	nitric oxide
NPs	nanoparticles
NSAIDs	non-steroidal anti-inflammatory drugs
NSCs	neural stem cells
OC	oxalyl chloride
OGD/R	oxygen–glucose deprivation/reoxygenation
PAK	p21-activated protein kinase
PDAs	polydopamine nanospheres
PLTs	platelets
PNSs	panax notoginseng total saponins
PS	phosphatidylcholine
PTT	photothermal therapy
Rac	Rac2-Tyr172
RAPA	rapamycin
RBC	red blood cell
Rg3	ginsenoside Rg3
ROS	reactive oxygen species
RvD1	resolvin D1
SHp	stroke-homing peptide
tPA	tissue plasminogen activator
TGF-β	transforming growth factor-beta
TNF-α	tumor necrosis factor-alpha
VEGF	vascular endothelial growth factor
VAV2	vav guanine nucleotide exchange factor 2
